# Reversal of stoma with biosynthetic mesh fascial reinforcement: a systematic review and meta‐analysis

**DOI:** 10.1111/codi.16913

**Published:** 2024-02-19

**Authors:** Dickson Dewantoro, Paul Manson, Miriam Brazzelli, George Ramsay

**Affiliations:** ^1^ Department of General Surgery Aberdeen Royal Infirmary Aberdeen UK; ^2^ Health Services Research Unit University of Aberdeen Aberdeen UK

**Keywords:** incisional hernia, mesh, mesh prophylaxis, stoma reversal

## Abstract

**Aim:**

Temporary stoma formation remains a common part of modern‐day colorectal surgical operations. At the time of reversal, a second procedure is required when the bowel is anastomosed and the musculature is closed. The rate of incisional hernia at these sites is 30%–35% with conventional suture closure. Mesh placement at this site is therefore an attractive option to reduce hernia risk, particularly as new mesh types, such as biosynthetic meshes, are available. The aim of this work was to conduct a systematic review and meta‐analysis assessing the use of mesh for prophylaxis of incisional hernia at stoma closure and to explore the outcome measures used by each of the included studies to establish whether they are genuinely patient‐centred.

**Method:**

This is a systematic review and meta‐analysis assessing the published literature regarding the use of mesh at stoma site closure operations. Comprehensive literature searches of major electronic databases were performed by an information specialist. Screening of search results was undertaken using standard systematic review principles. Data from selected studies were input into an Excel file. Meta‐analysis of the results of included studies was conducted using RevMan software (v.5.4). Randomized controlled trial (RCT) and non‐RCT data were analysed separately.

**Results:**

Eleven studies with a total of 2008 patients were selected for inclusion, with various mesh types used. Of the included studies, one was a RCT, seven were nonrandomized comparative studies and three were case series. The meta‐analysis of nonrandomized studies shows that the rate of incisional hernia was lower in the mesh reinforcement group compared with the suture closure group (OR 0.21, 95% CI 0.12–0.37) while rates of infection and haematoma/seroma were similar between groups (OR 0.7, 95% CI 0.41–1.21 and OR 1.05, 95% CI 0.63–1.80, respectively). The results of the RCT were in line with those of the nonrandomized studies.

**Conclusion:**

Current evidence indicates that mesh is safe and reduces incisional hernia. However, this is not commonly adopted into current clinical practice and the literature has minimal patient‐reported outcome measures. Future work should explore the reasons for such slow adoption as well as the preferences of patients in terms of outcome measures that matter most to them.

## INTRODUCTION

An intestinal stoma diverts the faecal stream onto the surface of the skin and away from the downstream colorectum. It remains a frequently performed part of complex colorectal surgery. In England and Wales, between 2014 and 2018, 6611 loop ileostomies were created for cancer alone [1]. Such stomas are often temporary and are closed (reversed) by a second procedure. However, the closure of the muscle and fascial layers at this second operation can be challenging, as identification of where the fascial sutures should sit can be technically difficult to achieve.

The rates of incisional hernia at this site can be as high as 30%–35% [2]. Such hernias are at risk of bowel obstruction or strangulation, which require an emergency operation. Indeed, 77% of these stoma site incisional hernias were symptomatic, with 65% requiring operative repair after 2 years of follow‐up in one cohort study [2]. Presentation of small bowel obstruction from abdominal wall hernia has an associated in‐hospital mortality rate of 9.4% [3]. Thus, an incisional hernia after stoma closure can lead to high‐risk complications.

The optimal method for fascia closure in stoma reversal procedures to prevent such incisional herniation has yet to be established and remains under debate [4, 5]. One potential intervention is to place a mesh at the time of stoma reversal. This aims to reinforce the abdominal wall, thereby providing prophylaxis of herniation. However, such an approach remains controversial. A stoma reversal site is, by definition, a contaminated one, as the bowel effluent has been sitting on this wound beforehand. As such, synthetic meshes, which are designed to be permanently present within the host tissue, may not be felt appropriate and could have unacceptably high postoperative infection rates [6]. Furthermore, their use is associated with concern amongst some patient groups [7].

Biological meshes, typically made of porcine collagen which degrades over time, are available and have been examined in the context of this operation [8, 9, 10]. However, these are expensive and take‐up of these products for this operation has been slow within colorectal practice. Biosynthetic meshes, which are slowly resorbed meshes that integrate into the tissues over time, have recently been developed for clinical use [11]. Biosynthetic meshes have the absorbable nature of a biological mesh without the high cost and have been demonstrated to be safe in contaminated fields and at stoma sites [6]. Even though these meshes are used for other indications, their use to reinforce the abdominal wall at the time of a stoma reversal in colorectal surgery has yet to be established.

While mesh for hernia prophylaxis may reduce the rates of incisional hernia, and hence the need for emergency surgery for bowel obstruction, the placement of a mesh may lead to problems such as erosion and infection. There is an additional question as to the optimal abdominal wall plane for placing such a mesh. Intraperitoneal, preperitoneal, sublay or onlay planes are all options in this context. Finally, it remains unclear as to which outcome measure in this field is most appropriate to report. The incisional hernia literature has been notoriously clinician focused, with diagnosis being established on clinical examination and by radiological assessment. It is unclear whether patient‐centred outcomes have been used in this research field.

Although meta‐analyses exploring the use of mesh at the site of stoma closure have previously been published [12], these were predominantly undertaken before the development of the new mesh types, which may have a crucial role in this clinical setting. An updated comprehensive review of the published literature in this field was, therefore, deemed to be of value. We aimed to conduct a systematic review and meta‐analysis assessing the use of mesh for prophylaxis of incisional hernia at stoma closure and to explore the outcome measures used by each of the included studies to establish whether they are genuinely patient‐centred.

## METHOD

### Study type

This is a systematic review and meta‐analysis assessing the impact of placing a mesh at the time of stoma closure on the rates of incisional hernia.

### Search strategy

A sensitive literature search strategy using database index terms and free text was developed by an information specialist (PM) to identify published peer‐reviewed studies. The databases searched were MEDLINE, EMBASE and the Cochrane Library (Figure S1). The searches focused on studies conducted in humans. There were no restrictions on study type, language, date or condition. The searches were carried out in June 2023. The search strategies are presented in Figure S1.

### Study selection

The population of interest was elective closure of colostomy or ileostomy operations. The intervention was mesh used for prophylaxis of hernia (any type of mesh placed in any anatomical plane). The comparison was suture closure and the outcome was incidence of incisional hernia (with particular focus on the definition of incisional hernia listed in each study). All other outcomes noted in each included paper were also noted. Particular care was taken to assess the relevance of outcomes from a patient perspective. Paediatric or emergency cases were excluded, as were studies on the use of mesh for treatment of incisional hernias at this site.

**FIGURE 1:**
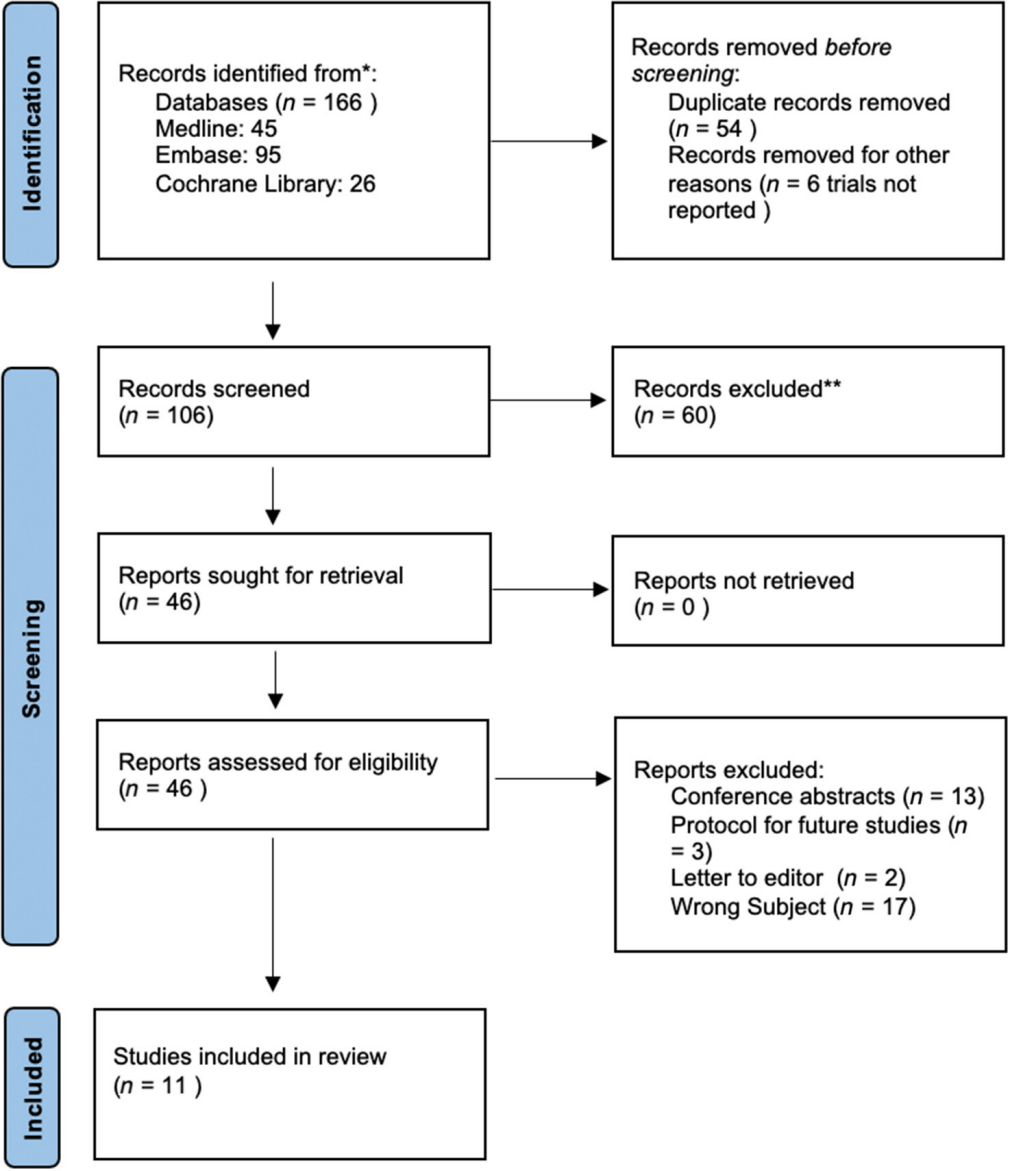
PRISMA graph.

Methods and results are reported in adherence with the Preferred Reporting Items for Systematic Reviews and Meta‐Analyses (PRISMA) Statement Criteria 2020. The PRISMA flowchart for study selection is shown in Figure 1.

### Data collection and analysis

Randomized clinical trials (RCTs) and nonrandomized studies (non‐RCTs), either prospective or retrospective, were deemed suitable for inclusion. RCT and non‐RCT data were kept separate for analysis. For each of the included studies we collected information on study design, demographic characteristics of participants, operative setting and procedure, type of mesh, anatomical site of mesh placement and outcome measures. Specific attention was paid to the time between mesh insertion and outcome assessment. Furthermore, patient‐centred outcome reporting, or lack thereof, was considered as a critical element to record.

Data were entered into an Excel spreadsheet (Microsoft, California, USA). Dichotomous outcome data (e.g. events of incisional hernia, infection rate) were combined in a random‐effects meta‐analysis using the Cochrane–Mantel–Haenszel method in Review Manager (RevMan v5.4). Estimates of effect were presented as odds ratios (ORs) with accompanied 95% confidence intervals (CIs). Statistical heterogeneity was quantified using *I* ^2^ statistics and the Cochrane *Q*‐test. Data from the single identified RCT are described narratively while data from comparative non‐RCTs are combined in a meta‐analysis.

### Risk of bias assessment

The risk of bias of non‐RCTs was assessed using an 18‐question checklist developed by the Health Services Research Unit at the University of Aberdeen in partnership with the Review Body for Interventional Procedures (ReBIP) at the National Institute for Health and Care Excellence. The questions covered the following aspects: generalizability, sample definition and selection, description of the intervention, outcome assessment, adequacy of follow‐up and performance of the analysis. Individual items were rated as ‘yes’, ‘no’ or ‘unclear’. A rating of ‘yes’ indicated a low risk of bias. The risk of bias of the included RCT was assessed using the Cochrane Risk of Bias tool (version 1) [13].

### Study registration

This work was registered with the International Prospective Register of Systematic Reviews (PROSPERO) database (number CRD 42022381308).

### Ethical review

As this was a review of published data and no new data were accrued, no ethical review was deemed necessary.

## RESULTS

The search strategies identified 166 citations of study reports. After removal of duplicates, 46 reports were retrieved for full‐text assessment. Two independent review authors (DD and GR) assessed all potentially relevant full‐text papers according to the predefined inclusion criteria. Any disagreement was settled through discussion or referred to a third review author (MB). Of the 46 potentially relevant studies identified by the search strategies, 11 were deemed suitable for inclusion [4, 6, 13, 14, 15, 16, 17, 18, 19, 20, 21]. Figure 1 shows the PRISMA flowchart for the selection process, including reasons for exclusion. Regarding the type of study design, we identified a single RCT, five prospective studies (two comparative cohort studies and three prospective case series) and five retrospective comparative cohort studies. The 11 included studies assessed a total of 2008 participants. Sample sizes varied across studies (range 7–790 participants). In eight studies, the primary endpoint was the rate of surgical site occurrence (SSO) or the rate of incisional hernia, while three studies assessed immediate postoperative complications. Across studies, none of the primary endpoints were patient‐reported outcome measures (PROMs). However, one study [4] included PROMs in its secondary analysis. Table 1 summarizes the characteristics of the included studies.

**TABLE 1:** Study types and outcomes assessed [4, 6, 13, 14, 15, 16, 17, 18, 19, 20, 21].

First author	Year of publication	DOI	Type of study	Population (*n*)	Mesh (*n*)	Suture closure (*n*)	Outcome measured	Outcome assessment	Other patient‐relevant outcomes
Liu [15]	2013	10.1007/s00268‐013‐2109‐3	Retrospective cohort study	83	83	47	Rate of stoma site herniation	Clinical or radiological	Time to hernia detection, rate of incisional hernia repair and stomal wound infection
Bhangu [13]	2014	10.1007/s10151‐013‐1001‐3	Prospective case series	7	7	0	Immediate postoperative outcome	Clinical	Wound infection, early hernia, reoperation
Maggiori [16]	2015	10.1016/j.surg.2015.07.004	Prospective comparative cohort study	94	94	30	Radiological hernia at 1 year after stomal reversal	Clinical and radiological	Wound infection (defined as a suppurative discharge at wound incision), postoperative morbidity (defined as any postoperative 30‐day or in‐hospital complication), clinical incisional hernia at 1 year (defined as a palpable fascial defect or visible protrusions at or near the surgical incision at rest or with Valsalva manoeuvre, and assessed in a blinded fashion) and a radiological parastomal hernia at the end of follow‐up
Warren [20]	2018	10.1016/j.surg.2017.09.041	Retrospective cohort study	359	359	91	Development of surgical site occurrence (wound complication not classified as a SSI, including seroma, haematoma, skin separation, skin necrosis, cellulitis or development of a chronic nonhealing wound), SSI, mesh‐related complications and hernia recurrence	Clinical and radiological	Duration of stay, readmission, operative time, blood loss and anastomotic leak rate
Bhangu [4]	2020	10.1016/S0140‐6736(19)32637‐6	Multicentre randomized controlled trial	790	373	386	Rate of clinically detected hernia at 2 years postrandomization	Clinical, radiological and symptomatic	Radiological hernia rate at 1 year postrandomization. Symptomatic hernia rate at 1 and 2 years postrandomization. This outcome measure was based on patient‐reported hernia symptoms including a local lump or pain at the site of the stoma closure. Surgical reintervention rates at 2 years postrandomization. Surgical complications, including SSIs (30 days postoperation and 1 year postrandomization) and seroma formation (1 year postrandomization). Quality of life assessed using EuroQolEQ‐5D (three‐level) at 30 days postoperation and 1 and 2 years postrandomization. Pain assessed using a 100‐point visual analogue scale at 30 days postoperation, and 1 and 2 years postrandomization. Health economic analysis, which was planned to be reported in a subsequent paper
Lee [14]	2020	PMID 32511101	Retrospective cohort study	33	33	18	Safety of the biological mesh defined as the need for mesh removal due to mesh‐related infectious complication	Clinical and radiological	Postoperative white cell count and C‐reactive protein, SSI, SSO, incisional hernia, morbidity and wound pain
Pizza [6]	2020	10.1007/s13304‐020‐00702‐z	Prospective comparative cohort study	84	84	26	Incidence of incisional hernia on side of LI following closure at 6 and 12 months after stomal reversal	Clinical and radiological	Incidence of wound events, morbidity and mortality of loop ileostomy closure and postoperative pain
Wong [21]	2020	10.1111/ans.15692	Retrospective cohort study	273	81	192	SSO	Clinical and radiological	NA
Tantawy [19]	2021	10.1093/qjmed/hcab097.047	Prospective case series	50	50	0	Detect the feasibility of application of prolene mesh at the site of stoma closure in reducing the rate of poststomal incisional hernia	Clinical	NA
Shaw [17]	2022	10.1007/s10029‐022‐02681‐z	Prospective case series	20	20	0	Assess whether prophylactic mesh reinforcement of LI closure was associated with increased rates of SSOs, including SSI (superficial, deep or organ space), dehiscence, seroma, haematoma, cellulitis or enterocutaneous fistula) compared with standard LI closure at 30 days postoperatively	Clinical and radiological	Evaluate the effectiveness of the prophylactic mesh implant at preventing incisional hernia formation
Siddiqui [18]	2023	10.1016/j.amjsurg.2023.04.013	Retrospective cohort study	215	67	148	Incisional hernia at the stoma site, development of SSO (wound complications not classified as SSI such as haematoma, seroma, cellulitis, skin necrosis or chronic nonhealing wounds) and SSIa	Serial clinical and radiological examination	Total operative time, estimated blood loss, hospital length of stay, readmission rate and need for reoperation

*Abbreviations*: LI, Loop Ileostomy; NA, Not assessed; SSI, surgical site infection; SSO, surgical site occurrence.

### Characteristics of the interventions

The primary operations (leading to the initial formation of the stoma) were predominantly for a cancer diagnosis. However, other reasons for the initial resection included diverticular disease, mesenteric ischaemia, sigmoid volvulus, trauma, inflammatory bowel disease and intestinal pseudo‐obstruction. All stoma closures were undertaken electively. Colostomies were created in 461 patients (185 with mesh placement and 276 in the suture closure cohort). Ileostomies were more frequent (*n* = 1547 patients, 643 with mesh insertion and 904 in the suture closure group). Comorbidities were reported in ten of the included studies and body mass index (BMI) assessment in eight studies. Across studies, there were variations in the type of mesh used. Two studies used a biological mesh, two studies a biosynthetic mesh and five used a synthetic mesh. The remaining two used a combination of biosynthetic and biological or synthetic and biological. There was also variation across studies in terms of the plane used for the placement of the mesh: five studies used onlay mesh, three retromuscular mesh, two intra‐abdominal/ peritoneal mesh and one either an onlay or retromuscular approach (see Table 2).

**TABLE 2:** Studies and types of mesh [4, 6, 13, 14, 15, 16, 17, 18, 19, 20, 21].

First author	Year of publication	Indication for stoma	Colostomy mesh (*n*)	Colostomy suture (*n*)	Ileostomy mesh (*n*)	Ileostomy suture (*n*)	BMI mesh (kg/m^2^)	BMI suture (kg/m^2^)	Comorbidities	Mesh type	Mesh plane
Liu [15]	2013	Cancer	0	0	47	36	25.6 ± 4.6	27.8 ± 5.3	Diabetes, hypertension, long‐term steroid use, CKD, respiratory condition, obesity (BMI >30), radiotherapy, chemotherapy	Synthetic	Onlay
Bhangu [13]	2014	Not mentioned	0	NA	7	NA	NA	NA	NA	Biological	Intra‐abdominal
Maggiori [16]	2015	Rectal cancer	0	0	30	64	26 ± 4 (range 19–36)	25 ± 4 (range 18–38)	Diabetes, COPD, preoperative radiotherapy, delay TME stoma reversal	Biosynthetic	Retromuscular
Warren [20]	2018	Not mentioned	67	145	24	123	30.2 ± 7.1	27.3 ± 6.4	Diabetes, COPD, hypertension, CAD, renal failure	Synthetic	Retromuscular
Bhangu [4]	2020	Cancer and other disease	0	0	15	18	22.7 ± 3.6	22.3 ± 3.2	Diabetes, hypertension, COPD, alcohol	Biological or synthetic	Onlay
Lee [14]	2020	Cancer and inflammatory bowel disease	0	0	26	58	24 ± 2 (range 20–38)	23 ± 3 (range 19–37)	Diabetes, COPD, heart ischaemia, oncological disease, inflammatory disease, neoadjuvant therapy	Biosynthetic	Onlay
Pizza [6]	2020	Cancer and other disease	79	80	315	316	26.8 ± 4.8	26.6 ± 5.2	Diabetes, steroid medication	Biological	Intra‐abdominal
Wong [21]	2020	Not stated	81	0	192	0	NA	NA	Not defined	Synthetic	Onlay
Tantawy [19]	2021	Cancer	0	NA	20	NA	29.19 ± 7.9	NA	Diabetes, steroids, COPD, neoadjuvant chemoradiotherapy, neoadjuvant therapy, adjuvant therapy	Synthetic	Retromuscular
Shaw [17]	2022	Bowel cacinoma, diverticular disease, mesenteric ischaemia, sigmoid volvulus, trauma, IBD, intestinal pseudo‐obstruction	20	NA	30	NA	NA	NA	Diabetes, infections, malignancy, liver or renal dysfunction, drug history	Synthetic	Onlay
Siddiqui [18]	2023	Both benign and malignant colorectal conditions	19	51	48	97	27.6 ± 5.3	28.7 ± 6.6	Diabetes, hypertension, COPD, coronary artery disease	Biosynthetic (*n* = 30), biological (*n* = 37)	Onlay (*n* = 27), retromuscular (*n* = 40)

*Abbreviations*: BMI, body mass index; CAD, coronary artery disease; CKD, chronic kidney disease; COPD, chronic obstructive pulmonary disease; IBD, inflammatory bowel disease; NA, Not assessed; TME, total mesorectal excision.

### Incisional hernia rates

**FIGURE 2:**
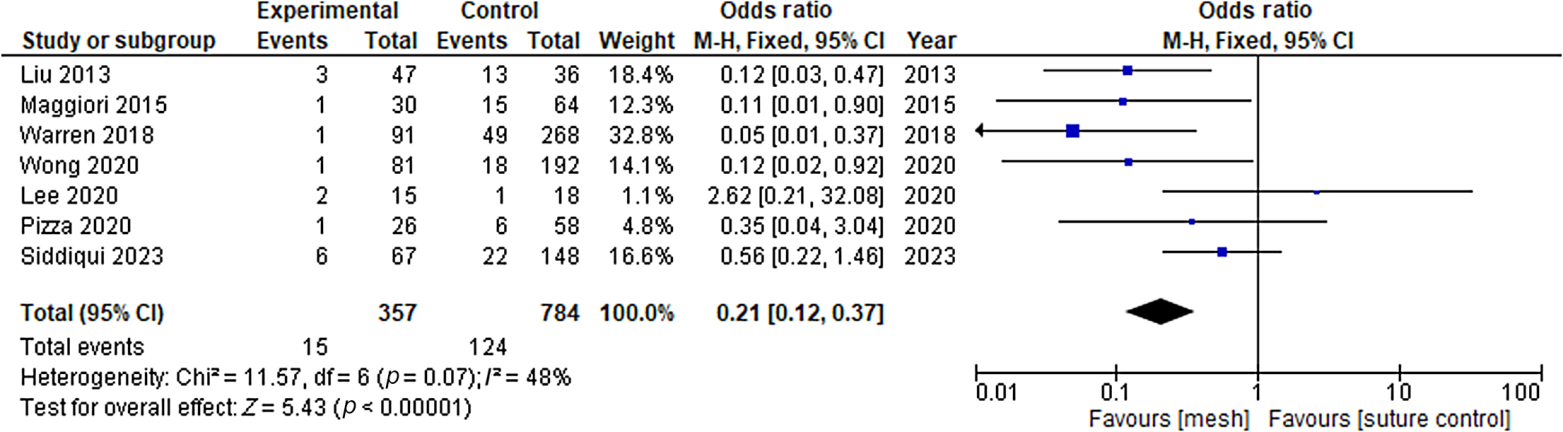
Incisional hernia rate.

Eight of the 11 included studies assessed mesh repairs versus suture repairs and reported data on the incidence of incisional hernia. Of these studies, one was a RCT and seven were comparative non‐RCTs. Nonrandomized data were combined in a meta‐analysis (see Figure 2). The rate of incisional hernia was significantly lower after mesh insertion compared with suture closure (OR 0.21, 95% CI 0.12–0.37; *p* < 0.0001). The cumulative percentage of events in the mesh group was 7.9% compared with 16.9% in the suture repair group. Similarly, the results of the single RCT show a lower incisional hernia rate at 2 years (Relative Risk [RR] 0.62, 95% CI 0.43–0.90; *p* = 0.012).

### Infection rate

**FIGURE 3:**
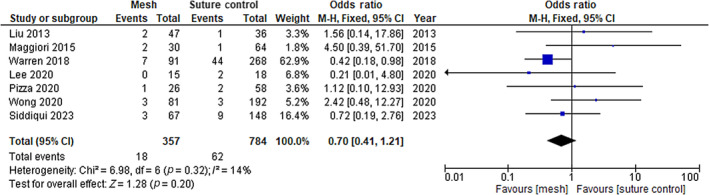
Infection rate.

The single RCT and seven non‐RCTs also reported data on the rate of infection. Meta‐analysis results are summarized in Figure 3. The rate of infection was not significantly different between groups (OR 0.7, 95% CI 0.41–1.2; *p* = 0.32). The cumulative percentage for mesh insertion was 7.8% compared with 9.0% for suture closure. The results of the included RCT are in line with those of the non‐RCTs (RR 1.19, 95% CI 0.84–1.68; *p* = 0.32).

### Haematoma/seroma

**FIGURE 4:**
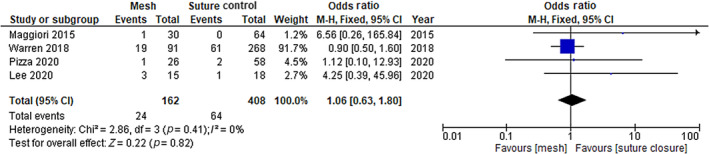
Haematoma or seroma rates.

Five studies (one RCT and four non‐RCTs) reported data on haematoma/ seroma. The rates of haematoma/seroma were not significantly different between the mesh placement group (5.0%) and suture closure (8.9%) group. The meta‐analysis of non‐RCTs showed an OR of 1.05 (95% CI 0.63–1.80; *p* = 0.41) (see Figure 4). Similarly, the RCT reported a RR of 1·26 (95% CI 0.51–3.14; *p* = 0.61).

### Reoperation

**FIGURE 5:**
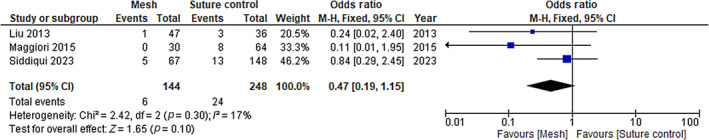
Reoperation rates.

Four studies (one RCT and three non‐RCTs) reported data on reoperation rates (in the short term after the stoma closure). Figure 5 shows a forest plot of the meta‐analysis results. There was no significant difference between mesh repair and suture closure in terms of rate of reoperation (3.9% vs. 6.6%, respectively; OR 0.47, 95% CI 0.19–1.15; *p* = 0.3). Similarly, the results of the RCT showed a RR of 0·78 (95% CI 0.54–1.13; *p* = 0.19).

### Patient‐reported outcome measures

Only the included RCT provided data on PROMs [4]. Quality of life was assessed using the EQ5D EuroQal and ED5D VAS scores. Interestingly, no differences were observed between participants treated with mesh repair and those treated with suture repair at 30 days, 1 and 2 years after surgery.

### Risk of bias assessment

The single RCT was deemed to be at low risk of bias across all domains. Most of the non‐RCTs were judged at high risk of bias due to the lack of randomization and selection and recall biases. The results of the risk of bias assessment are included in Figure S2.

## DISCUSSION

We have conducted a comprehensive systematic review of the published literature assessing the use of mesh at the time of stoma closure for the prophylaxis of incisional hernia in elective colorectal surgery. Our meta‐analysis results indicate that the use of mesh may reduce the risk of incisional hernia without increasing the rates of surgical site infection, haematoma, seroma or short‐term reoperation. However, the current evidence base is limited to a few studies of poor methodological quality. Compared with previous published systematic reviews, we identified one RCT conducted in this clinical area.

It is also worth noting that all included studies focused on clinician‐relevant outcomes. Each had either radiological identification of hernia at set time points after stoma closure or hernia diagnosis at the time of clinical examination. Only one study [4] assessed patient‐reported quality of life as a secondary endpoint. This emphasizes the lack of patient‐reported outcome data in the field of hernia surgery [22]. It is also striking that, in this study, no difference in quality of life, as assessed by EQ5D, was noted with the use of mesh or suture repair. Given that this is a single study, this result is difficult to interpret. Firstly, the use of mesh did not adversely affect the quality of life in this cohort. However, no improvement in quality of life was noted with the identified reduction in the rate of incisional hernia development. Thus, future works must concentrate more on patient‐identified outcomes rather than those seen through the viewpoint of a clinician. At present, there is no agreed core outcome set for incisional hernia repair. However, the HarMoNy project aims to develop such a core outcome set in incisional hernia and will help address this issue in the near future [23].

The studies included in this systematic review varied in terms of study design, methodological quality and sample size. The ROCCS study, which assessed 790 participants and used biological mesh in the intra‐abdominal plane of the abdominal wall, is the only RCT in this field [4]. It demonstrates the safety of the mesh technique and a reduction in occurrence at the surgical site. These findings are supplemented by data from comparative cohort studies identified in the literature. Nevertheless, at present, mesh placement at the time of stoma closure is not a uniform practice in colorectal surgery. Any future study would need to explore why surgeons are reluctant to use these techniques in clinical practice. Canvassing the opinion of surgeons around what would be required for implementing a change of practice would be an important step in moving this clinical field forward.

Future research would also need to explore whether such an approach would be acceptable to patients. Complications associated with incisional hernia [24, 25] can cause significant chronic pain in some patients, with a consequent reduction in quality of life. However, in most instances the use of mesh has been proposed for the treatment of established hernias rather than for hernia prophylaxis. Future studies should consider the involvement of patient partners and key stakeholders to decide whether the use of mesh is acceptable in this setting and whether it should be used uniformly or in the identification and selection of high‐risk groups. Discrete choice experiment designs could be used to enquire which risk profile would be acceptable to patients to reduce the potential risk of incisional herniation.

A further area that requires consideration is the choice of mesh and the site of placement. A synthetic mesh was used in most studies [4, 15, 17, 19, 20] while a biological mesh was used only in the identified RCT [4]. There have been only three small studies to date that used biosynthetic mesh types [14, 16, 18]. These were all included in this analysis, with a total of 157 patients with biosynthetic mesh placement. Furthermore, the current evidence is insufficient to determine the optimal implant and plane. The ROCCS trial used the intra‐abdominal plane, which requires some clinical training to perform the procedure well. Mesh placement within the abdomen is likely to require the use of the more expensive biological meshes whereas for other planes any mesh type would be suitable. Additionally, in the PRIMA study the onlay plane was demonstrated to be effective in hernia prophylaxis in the midline [26].

### Strengths and weaknesses

To our knowledge, this is the most complete systematic review and meta‐analysis to assess the prophylactic use of meshes for the prevention of incisional hernia after a stoma. Compared with previously published systematic reviews on this topic [12], our meta‐analysis includes the only assessment of PROMs. This work also highlights the clinical questions that remained to be addressed. At present there is a dearth of high‐quality RCTs. Furthermore, the quality of data available from prospective and retrospective non‐RCTs varies and is generally poor. It is also difficult to fathom why the use of prophylactic meshes, which do not seem to increase the rate of infection and may be effective in reducing the risk of incisional hernia, have not been investigated and adopted more widely in standard colorectal practice. Future research is needed and should include work with patient groups to ensure that any future study addresses and covers issues that are important to patients.

## CONCLUSION

The prophylactic use of mesh may reduce the occurrence of incisional hernia without increasing rates of infection, haematoma or seroma. Future research needs to explore patients’ and surgeons’ perceptions of these techniques to inform well‐designed RCTs. The current literature focuses primarily on clinical outcomes and does not give much consideration to patient‐reported outcomes. Future studies should determine which is the optimal type of mesh and plane for the prevention of hernia recurrence as well as assess outcomes that matter most to patients.

## AUTHOR CONTRIBUTIONS

**Dickson Dewantoro:** Conceptualization; methodology; data curation; formal analysis; visualization; writing – original draft; writing – review and editing. **Paul Manson:** Resources; writing – review and editing; validation; investigation; data curation; software; project administration. **Miriam Brazzelli:** Conceptualization; methodology; writing – review and editing; writing – original draft; validation; formal analysis. **George Ramsay:** Conceptualization; methodology; software; data curation; investigation; validation; formal analysis; supervision; funding acquisition; visualization; project administration; resources; writing – original draft; writing – review and editing.

## FUNDING INFORMATION

This study was funded by ISSF Wellcome Trust University of Aberdeen Seedcorn Grant.

## CONFLICT OF INTEREST STATEMENT

There were no conflict of interests to declare.

## ETHICAL STATEMENT

This was a systematic review/meta‐analysis of previously published literature and so no further ethical review was sought.

## Supporting information


Figure S1.

Figure S2.

## Data Availability

The data that support the findings of this study are available from the corresponding author upon reasonable request.
