# Supplementary material for: Reversal of stoma with biosynthetic mesh fascial reinforcement: a systematic review and meta‐analysis
Source: Colorectal Dis. 2024 Feb 19;26(4):632–42. doi: 10.1111/codi.16913 (PMC12150827; doi:10.1111/codi.16913)
Supplement: Supplementary file 1 — Figure S1. [file CODI-26-632-s001.docx]

**Supplementary figure 1: Search terms**

**Ovid MEDLINE(R) and Epub Ahead of Print, In-Process, In-Data-Review & Other Non-Indexed Citations, Daily and Versions <1946 to June 9, 2023>**

1 Ileostomy/ 7189

2 Colostomy/ 9300

3 1 or 2 14821

4 (revers* or clos*).tw. 1830858

5 3 and 4 2249

6 ((revers* or clos*) adj4 (ileostom* or colostom*)).tw. 2217

7 (((revers* or clos*) adj4 (enterostom* or ostom* or stoma?)) and (ilesostom* or colostom*)).tw. 302

8 5 or 6 or 7 3328

9 Surgical Mesh/ 16174

10 (mesh or meshes).tw. 45369

11 9 or 10 49801

12 Incisional Hernia/ 1249

13 hernia/ 8349

14 incisional.tw. 12492

15 13 and 14 257

16 ((incisional adj4 hernia?) or S?IH).tw. 8369

17 12 or 15 or 16 8694

18 8 and 11 and 17 43

**Embase <1974 to 2022 Week 41>**

1 ileostomy/ 16114

2 colostomy/ 16854

3 1 or 2 29896

4 (revers* or clos*).tw. 2194659

5 3 and 4 5879

6 ((revers* or clos*) adj4 (ileostom* or colostom*)).tw. 3488

7 (((revers* or clos*) adj4 (enterostom* or ostom* or stoma?)) and (ilesostom* or colostom*)).tw. 517

8 5 or 6 or 7 6451

9 surgical mesh/ 10048

10 (mesh or meshes).tw. 63425

11 9 or 10 66974

12 incisional hernia/ 8556

13 hernia/ 18303

14 incisional.tw. 17930

15 13 and 14 862

16 ((incisional adj4 hernia?) or S?IH).tw. 11230

17 12 or 15 or 16 14083

18 8 and 11 and 17 84

**Cochrane Library**

ID Search Hits

#1 MeSH descriptor: [Ileostomy] this term only 219

#2 MeSH descriptor: [Colostomy] this term only 189

#3 #1 or #2 370

#4 revers* or clos* 85810

#5 #3 and #4 119

#6 (revers* or clos*) Near/4 (ileostom* or colostom*) 337

#7 ((revers* or clos*) Near/4 (enterostom* or ostom* or stoma?)) and (ilesostom* or colostom*) 71

#8 #5 or #6 or #7 399

#9 MeSH descriptor: [Surgical Mesh] this term only 838

#10 mesh or meshes 9655

#11 #9 or #10 9655

#12 MeSH descriptor: [Incisional Hernia] this term only 166

#13 MeSH descriptor: [Hernia] this term only 627

#14 incisional 2281

#15 #13 and #14 123

#16 (incisional Near/4 hernia?) or S?IH 2403

#17 #12 or #15 or #16 2408

#18 #8 and #11 and #17 20
