# Supplementary material for: Reversal of stoma with biosynthetic mesh fascial reinforcement: a systematic review and meta‐analysis
Source: Colorectal Dis. 2024 Feb 19;26(4):632–42. doi: 10.1111/codi.16913 (PMC12150827; doi:10.1111/codi.16913)
Supplement: Supplementary file 2 — Figure S2. [file CODI-26-632-s002.docx]

**Supplementary figure 2: Risk of Bias assessment**

Lui 2013 Date evaluated: 13/6/2023

Study ID: 10.1007/s00268-013-2109-3

| **Criteria** | **Yes** | **No** | **Unclear** | **Comments** |
| --- | --- | --- | --- | --- |
| 1. Were participants a representative sample selected from a relevant patient population, e.g. randomly selected from those seeking for treatment despite of age, duration of disease, primary or secondary disease, and severity of disease? | x |  |  |  |
| 1. Were the inclusion/exclusion criteria of participants clearly described? | x |  |  |  |
| 1. Were participants entering the study at a similar point in their disease progression, i.e. severity of disease? |  |  | x |  |
| 1. Was selection of patients consecutive? |  |  | x |  |
| 1. Was data collection undertaken prospectively? |  | x |  | Retrospective |
| 1. *Were the groups comparable on demographic characteristics and clinical features?* | x |  |  |  |
| 1. Was the intervention (and comparison) clearly defined? | x |  |  |  |
| 1. Was the intervention undertaken by someone experienced at performing the procedure?^1^ | x |  |  |  |
| 1. Were the staff, place, and facilities where the patients were treated appropriate for performing the procedure? (E.g. access to back-up facilities in hospital or special clinic) |  |  | x |  |
| 1. Were any of the important outcomes considered, i.e. on clinical effectiveness, cost-effectiveness, or learning curves? | x |  |  |  |
| 1. Were objective (valid and reliable) outcome measures used, including satisfaction scale? | x |  |  |  |
| 1. *Was the assessment of main outcomes blind?* |  | x |  |  |
| 1. Was follow-up long enough (>=1y) to detect important effects on outcomes of interest? | x |  |  |  |
| 1. Was information provided on non-respondents, dropouts?^2^ |  | x |  |  |
| 1. Were the withdrawals/drop-outs having similar characteristics as those completed the study and therefore unlikely to cause bias?^3^ |  |  | x |  |
| 1. *Was length of follow-up similar between comparison groups* | x |  |  |  |
| 1. Were the important prognostic factors identified, e.g. age, duration of disease, disease severity?^4^ | x |  |  |  |
| 1. *Were the analyses adjusted for confounding factors?* | x |  |  |  |

The same form was adapted to assess the quality of case series after taking out question 6, 12, 16 and 18.

Note:

1. ‘Yes’ if the practitioner received training on conducting the procedure before or conducted same kind of procedure before, i.e. no learning curve.

2. ‘No’ if participants were from those whose follow up records were available (retrospective)

3. ‘Yes’ if no withdrawal/drop out; ‘No’ if drop-out rate >=30% or differential drop-out, e.g. those having most severe disease died during follow up but the death was not due to treatment; no description of those lost. 4. ‘Yes’ if two or more than two factors were identified.

Bhangu 2014 Date evaluated: 13/6/2023

Study ID: 10.1007/s00268-013-2109-3

| **Criteria** | **Yes** | **No** | **Unclear** | **Comments** |
| --- | --- | --- | --- | --- |
| 1. Were participants a representative sample selected from a relevant patient population, e.g. randomly selected from those seeking for treatment despite of age, duration of disease, primary or secondary disease, and severity of disease? | x |  |  |  |
| 1. Were the inclusion/exclusion criteria of participants clearly described? | x |  |  |  |
| 1. Were participants entering the study at a similar point in their disease progression, i.e. severity of disease? |  |  | x |  |
| 1. Was selection of patients consecutive? |  |  | x |  |
| 1. Was data collection undertaken prospectively? |  | x |  | Retrospective |
| 1. *Were the groups comparable on demographic characteristics and clinical features?* | x |  |  |  |
| 1. Was the intervention (and comparison) clearly defined? | x |  |  |  |
| 1. Was the intervention undertaken by someone experienced at performing the procedure?^1^ | x |  |  |  |
| 1. Were the staff, place, and facilities where the patients were treated appropriate for performing the procedure? (E.g. access to back-up facilities in hospital or special clinic) |  |  | x |  |
| 1. Were any of the important outcomes considered, i.e. on clinical effectiveness, cost-effectiveness, or learning curves? | x |  |  |  |
| 1. Were objective (valid and reliable) outcome measures used, including satisfaction scale? | x |  |  |  |
| 1. *Was the assessment of main outcomes blind?* |  | x |  |  |
| 1. Was follow-up long enough (>=1y) to detect important effects on outcomes of interest? | x |  |  |  |
| 1. Was information provided on non-respondents, dropouts?^2^ |  | x |  |  |
| 1. Were the withdrawals/drop-outs having similar characteristics as those completed the study and therefore unlikely to cause bias?^3^ |  |  | x |  |
| 1. *Was length of follow-up similar between comparison groups* | x |  |  |  |
| 1. Were the important prognostic factors identified, e.g. age, duration of disease, disease severity?^4^ | x |  |  |  |
| 1. *Were the analyses adjusted for confounding factors?* | x |  |  |  |

The same form was adapted to assess the quality of case series after taking out question 6, 12, 16 and 18.

Note:

1. ‘Yes’ if the practitioner received training on conducting the procedure before or conducted same kind of procedure before, i.e. no learning curve.

2. ‘No’ if participants were from those whose follow up records were available (retrospective)

3. ‘Yes’ if no withdrawal/drop out; ‘No’ if drop-out rate >=30% or differential drop-out, e.g. those having most severe disease died during follow up but the death was not due to treatment; no description of those lost.

4. ‘Yes’ if two or more than two factors were identified.

Maggiori 2015 * Date evaluated: 13/6/2023

Study ID: [10.1016/j.surg.2015.07.004](https://doi.org/10.1016/j.surg.2015.07.004)

| **Criteria** | **Yes** | **No** | **Unclear** | **Comments** |
| --- | --- | --- | --- | --- |
| 1. Were participants a representative sample selected from a relevant patient population, e.g. randomly selected from those seeking for treatment despite of age, duration of disease, primary or secondary disease, and severity of disease? | X |  |  |  |
| 1. Were the inclusion/exclusion criteria of participants clearly described? | X |  |  |  |
| 1. Were participants entering the study at a similar point in their disease progression, i.e. severity of disease? | X |  |  |  |
| 1. Was selection of patients consecutive? | X |  |  |  |
| 1. Was data collection undertaken prospectively? | X |  |  |  |
| 1. *Were the groups comparable on demographic characteristics and clinical features?* | X |  |  |  |
| 1. Was the intervention (and comparison) clearly defined? | X |  |  |  |
| 1. Was the intervention undertaken by someone experienced at performing the procedure?^1^ | X |  |  |  |
| 1. Were the staff, place, and facilities where the patients were treated appropriate for performing the procedure? (E.g. access to back-up facilities in hospital or special clinic) |  |  | X |  |
| 1. Were any of the important outcomes considered, i.e. on clinical effectiveness, cost-effectiveness, or learning curves? | X |  |  |  |
| 1. Were objective (valid and reliable) outcome measures used, including satisfaction scale? | X |  |  |  |
| 1. *Was the assessment of main outcomes blind?* | X |  |  |  |
| 1. Was follow-up long enough (>=1y) to detect important effects on outcomes of interest? | X |  |  |  |
| 1. Was information provided on non-respondents, dropouts?^2^ |  | X |  |  |
| 1. Were the withdrawals/drop-outs having similar characteristics as those completed the study and therefore unlikely to cause bias?^3^ | X |  |  |  |
| 1. *Was length of follow-up similar between comparison groups* | X |  |  |  |
| 1. Were the important prognostic factors identified, e.g. age, duration of disease, disease severity?^4^ | X |  |  |  |
| 1. *Were the analyses adjusted for confounding factors?* | X |  |  |  |

The same form was adapted to assess the quality of case series after taking out question 6, 12, 16 and 18.

Note:

1. ‘Yes’ if the practitioner received training on conducting the procedure before or conducted same kind of procedure before, i.e. no learning curve.

2. ‘No’ if participants were from those whose follow up records were available (retrospective)

3. ‘Yes’ if no withdrawal/drop out; ‘No’ if drop-out rate >=30% or differential drop-out, e.g. those having most severe disease died during follow up but the death was not due to treatment; no description of those lost.

4. ‘Yes’ if two or more than two factors were identified.

Warren 2018 Date evaluated: 13/06/2023

Study ID: 10.1016/j.surg.2017.09.041

| **Criteria** | **Yes** | **No** | **Unclear** | **Comments** |
| --- | --- | --- | --- | --- |
| 1. Were participants a representative sample selected from a relevant patient population, e.g. randomly selected from those seeking for treatment despite of age, duration of disease, primary or secondary disease, and severity of disease? | X |  |  |  |
| 1. Were the inclusion/exclusion criteria of participants clearly described? | X |  |  |  |
| 1. Were participants entering the study at a similar point in their disease progression, i.e. severity of disease? |  |  | X |  |
| 1. Was selection of patients consecutive? | X |  |  |  |
| 1. Was data collection undertaken prospectively? |  | X |  |  |
| 1. *Were the groups comparable on demographic characteristics and clinical features?* | X |  |  |  |
| 1. Was the intervention (and comparison) clearly defined? | X |  |  |  |
| 1. Was the intervention undertaken by someone experienced at performing the procedure?^1^ | X |  |  |  |
| 1. Were the staff, place, and facilities where the patients were treated appropriate for performing the procedure? (E.g. access to back-up facilities in hospital or special clinic) |  |  | X |  |
| 1. Were any of the important outcomes considered, i.e. on clinical effectiveness, cost-effectiveness, or learning curves? | X |  |  |  |
| 1. Were objective (valid and reliable) outcome measures used, including satisfaction scale? | X |  |  |  |
| 1. *Was the assessment of main outcomes blind?* |  | X |  |  |
| 1. Was follow-up long enough (>=1y) to detect important effects on outcomes of interest? | X |  |  |  |
| 1. Was information provided on non-respondents, dropouts?^2^ |  | X |  |  |
| 1. Were the withdrawals/drop-outs having similar characteristics as those completed the study and therefore unlikely to cause bias?^3^ | X |  |  |  |
| 1. *Was length of follow-up similar between comparison groups* |  | X |  |  |
| 1. Were the important prognostic factors identified, e.g. age, duration of disease, disease severity?^4^ | X |  |  |  |
| 1. *Were the analyses adjusted for confounding factors?* | X |  |  |  |

The same form was adapted to assess the quality of case series after taking out question 6, 12, 16 and 18.

Note:

1. ‘Yes’ if the practitioner received training on conducting the procedure before or conducted same kind of procedure before, i.e. no learning curve.

2. ‘No’ if participants were from those whose follow up records were available (retrospective)

3. ‘Yes’ if no withdrawal/drop out; ‘No’ if drop-out rate >=30% or differential drop-out, e.g. those having most severe disease died during follow up but the death was not due to treatment; no description of those lost.

4. ‘Yes’ if two or more than two factors were identified.

Bhangu 2020 Date evaluated: 13/6/2023

Study ID: 10.1016/S0140-6736(19)32637-6

Lee 2020 Date evaluated: 10/06/2023

Study ID: PMID: 32511101

| **Criteria** | **Yes** | **No** | **Unclear** | **Comments** |
| --- | --- | --- | --- | --- |
| 1. Were participants a representative sample selected from a relevant patient population, e.g. randomly selected from those seeking for treatment despite of age, duration of disease, primary or secondary disease, and severity of disease? |  |  | X |  |
| 1. Were the inclusion/exclusion criteria of participants clearly described? | X |  |  |  |
| 1. Were participants entering the study at a similar point in their disease progression, i.e. severity of disease? |  |  | X |  |
| 1. Was selection of patients consecutive? | X |  |  |  |
| 1. Was data collection undertaken prospectively? |  | X |  |  |
| 1. *Were the groups comparable on demographic characteristics and clinical features?* | X |  |  |  |
| 1. Was the intervention (and comparison) clearly defined? | X |  |  |  |
| 1. Was the intervention undertaken by someone experienced at performing the procedure?^1^ | X |  |  |  |
| 1. Were the staff, place, and facilities where the patients were treated appropriate for performing the procedure? (E.g. access to back-up facilities in hospital or special clinic) |  |  | X |  |
| 1. Were any of the important outcomes considered, i.e. on clinical effectiveness, cost-effectiveness, or learning curves? | X |  |  |  |
| 1. Were objective (valid and reliable) outcome measures used, including satisfaction scale? | X |  |  |  |
| 1. *Was the assessment of main outcomes blind?* |  | X |  |  |
| 1. Was follow-up long enough (>=1y) to detect important effects on outcomes of interest? | X |  |  |  |
| 1. Was information provided on non-respondents, dropouts?^2^ |  | X |  |  |
| 1. Were the withdrawals/drop-outs having similar characteristics as those completed the study and therefore unlikely to cause bias?^3^ |  |  |  | NA |
| 1. *Was length of follow-up similar between comparison groups* | X |  |  |  |
| 1. Were the important prognostic factors identified, e.g. age, duration of disease, disease severity?^4^ | X |  |  |  |
| 1. *Were the analyses adjusted for confounding factors?* | X |  |  |  |

The same form was adapted to assess the quality of case series after taking out question 6, 12, 16 and 18.

Note:

1. ‘Yes’ if the practitioner received training on conducting the procedure before or conducted same kind of procedure before, i.e. no learning curve.

2. ‘No’ if participants were from those whose follow up records were available (retrospective)

3. ‘Yes’ if no withdrawal/drop out; ‘No’ if drop-out rate >=30% or differential drop-out, e.g. those having most severe disease died during follow up but the death was not due to treatment; no description of those lost.

4. ‘Yes’ if two or more than two factors were identified.

Pizza 2020 Date evaluated: 13/6/2023

Study ID: 0.1007/s13304-020-00702-z

| **Criteria** | **Yes** | **No** | **Unclear** | **Comments** |
| --- | --- | --- | --- | --- |
| 1. Were participants a representative sample selected from a relevant patient population, e.g. randomly selected from those seeking for treatment despite of age, duration of disease, primary or secondary disease, and severity of disease? | X |  |  |  |
| 1. Were the inclusion/exclusion criteria of participants clearly described? | X |  |  |  |
| 1. Were participants entering the study at a similar point in their disease progression, i.e. severity of disease? |  |  | X |  |
| 1. Was selection of patients consecutive? | X |  |  |  |
| 1. Was data collection undertaken prospectively? | X |  |  |  |
| 1. *Were the groups comparable on demographic characteristics and clinical features?* | X |  |  |  |
| 1. Was the intervention (and comparison) clearly defined? | X |  |  |  |
| 1. Was the intervention undertaken by someone experienced at performing the procedure?^1^ | X |  |  |  |
| 1. Were the staff, place, and facilities where the patients were treated appropriate for performing the procedure? (E.g. access to back-up facilities in hospital or special clinic) |  |  | X |  |
| 1. Were any of the important outcomes considered, i.e. on clinical effectiveness, cost-effectiveness, or learning curves? | X |  |  |  |
| 1. Were objective (valid and reliable) outcome measures used, including satisfaction scale? | X |  |  |  |
| 1. *Was the assessment of main outcomes blind?* |  | X |  |  |
| 1. Was follow-up long enough (>=1y) to detect important effects on outcomes of interest? | X |  |  |  |
| 1. Was information provided on non-respondents, dropouts?^2^ |  | X |  |  |
| 1. Were the withdrawals/drop-outs having similar characteristics as those completed the study and therefore unlikely to cause bias?^3^ |  |  | X |  |
| 1. *Was length of follow-up similar between comparison groups* | X |  |  |  |
| 1. Were the important prognostic factors identified, e.g. age, duration of disease, disease severity?^4^ | X |  |  |  |
| 1. *Were the analyses adjusted for confounding factors?* | X |  |  |  |

The same form was adapted to assess the quality of case series after taking out question 6, 12, 16 and 18.

Note:

1. ‘Yes’ if the practitioner received training on conducting the procedure before or conducted same kind of procedure before, i.e. no learning curve.

2. ‘No’ if participants were from those whose follow up records were available (retrospective)

3. ‘Yes’ if no withdrawal/drop out; ‘No’ if drop-out rate >=30% or differential drop-out, e.g. those having most severe disease died during follow up but the death was not due to treatment; no description of those lost.

4. ‘Yes’ if two or more than two factors were identified.

Wong 2020 Date evaluated: 13/6/2023

Study ID: 10.1111/ans.15692

| **Criteria** | **Yes** | **No** | **Unclear** | **Comments** |
| --- | --- | --- | --- | --- |
| 1. Were participants a representative sample selected from a relevant patient population, e.g. randomly selected from those seeking for treatment despite of age, duration of disease, primary or secondary disease, and severity of disease? | X |  |  |  |
| 1. Were the inclusion/exclusion criteria of participants clearly described? | X |  |  |  |
| 1. Were participants entering the study at a similar point in their disease progression, i.e. severity of disease? |  |  | X |  |
| 1. Was selection of patients consecutive? |  |  | X |  |
| 1. Was data collection undertaken prospectively? |  | X |  |  |
| 1. *Were the groups comparable on demographic characteristics and clinical features?* | X |  |  |  |
| 1. Was the intervention (and comparison) clearly defined? | X |  |  |  |
| 1. Was the intervention undertaken by someone experienced at performing the procedure?^1^ | X |  |  |  |
| 1. Were the staff, place, and facilities where the patients were treated appropriate for performing the procedure? (E.g. access to back-up facilities in hospital or special clinic) |  |  | X |  |
| 1. Were any of the important outcomes considered, i.e. on clinical effectiveness, cost-effectiveness, or learning curves? | X |  |  |  |
| 1. Were objective (valid and reliable) outcome measures used, including satisfaction scale? | X |  |  |  |
| 1. *Was the assessment of main outcomes blind?* | X |  |  |  |
| 1. Was follow-up long enough (>=1y) to detect important effects on outcomes of interest? | X |  |  |  |
| 1. Was information provided on non-respondents, dropouts?^2^ |  | X |  |  |
| 1. Were the withdrawals/drop-outs having similar characteristics as those completed the study and therefore unlikely to cause bias?^3^ |  |  |  | NA |
| 1. *Was length of follow-up similar between comparison groups* | X |  |  |  |
| 1. Were the important prognostic factors identified, e.g. age, duration of disease, disease severity?^4^ | X |  |  |  |
| 1. *Were the analyses adjusted for confounding factors?* | X |  |  |  |

The same form was adapted to assess the quality of case series after taking out question 6, 12, 16 and 18.

Note:

1. ‘Yes’ if the practitioner received training on conducting the procedure before or conducted same kind of procedure before, i.e. no learning curve.

2. ‘No’ if participants were from those whose follow up records were available (retrospective)

3. ‘Yes’ if no withdrawal/drop out; ‘No’ if drop-out rate >=30% or differential drop-out, e.g. those having most severe disease died during follow up but the death was not due to treatment; no description of those lost.

4. ‘Yes’ if two or more than two factors were identified.

Tantawy 2021 Date evaluated: 13/6/2023

Study ID: [10.1093/qjmed/hcab097.047](https://doi.org/10.1093/qjmed/hcab097.047)

| **Criteria** | **Yes** | **No** | **Unclear** | **Comments** |
| --- | --- | --- | --- | --- |
| 1. Were participants a representative sample selected from a relevant patient population, e.g. randomly selected from those seeking for treatment despite of age, duration of disease, primary or secondary disease, and severity of disease? |  |  | X |  |
| 1. Were the inclusion/exclusion criteria of participants clearly described? | X |  |  |  |
| 1. Were participants entering the study at a similar point in their disease progression, i.e. severity of disease? |  |  | X |  |
| 1. Was selection of patients consecutive? | X |  |  |  |
| 1. Was data collection undertaken prospectively? | X |  |  |  |
| 1. *Were the groups comparable on demographic characteristics and clinical features?* |  |  | X |  |
| 1. Was the intervention (and comparison) clearly defined? | X |  |  |  |
| 1. Was the intervention undertaken by someone experienced at performing the procedure?^1^ | X |  |  |  |
| 1. Were the staff, place, and facilities where the patients were treated appropriate for performing the procedure? (E.g. access to back-up facilities in hospital or special clinic) |  |  | X |  |
| 1. Were any of the important outcomes considered, i.e. on clinical effectiveness, cost-effectiveness, or learning curves? | X |  |  |  |
| 1. Were objective (valid and reliable) outcome measures used, including satisfaction scale? | X |  |  |  |
| 1. *Was the assessment of main outcomes blind?* |  | X |  |  |
| 1. Was follow-up long enough (>=1y) to detect important effects on outcomes of interest? |  | X |  |  |
| 1. Was information provided on non-respondents, dropouts?^2^ |  | X |  |  |
| 1. Were the withdrawals/drop-outs having similar characteristics as those completed the study and therefore unlikely to cause bias?^3^ |  |  | X |  |
| 1. *Was length of follow-up similar between comparison groups* | X |  |  |  |
| 1. Were the important prognostic factors identified, e.g. age, duration of disease, disease severity?^4^ | X |  |  |  |
| 1. *Were the analyses adjusted for confounding factors?* |  |  | X |  |

The same form was adapted to assess the quality of case series after taking out question 6, 12, 16 and 18.

Note:

1. ‘Yes’ if the practitioner received training on conducting the procedure before or conducted same kind of procedure before, i.e. no learning curve.

2. ‘No’ if participants were from those whose follow up records were available (retrospective)

3. ‘Yes’ if no withdrawal/drop out; ‘No’ if drop-out rate >=30% or differential drop-out, e.g. those having most severe disease died during follow up but the death was not due to treatment; no description of those lost.

4. ‘Yes’ if two or more than two factors were identified.

Shaw 2022 Date evaluated: 13/6/2023

Study ID: [10.1007/s10029-022-02681-z](https://doi.org/10.1007/s10029-022-02681-z)

| **Criteria** | **Yes** | **No** | **Unclear** | **Comments** |
| --- | --- | --- | --- | --- |
| 1. Were participants a representative sample selected from a relevant patient population, e.g. randomly selected from those seeking for treatment despite of age, duration of disease, primary or secondary disease, and severity of disease? | X |  |  |  |
| 1. Were the inclusion/exclusion criteria of participants clearly described? | X |  |  |  |
| 1. Were participants entering the study at a similar point in their disease progression, i.e. severity of disease? | X |  |  |  |
| 1. Was selection of patients consecutive? |  |  | X |  |
| 1. Was data collection undertaken prospectively? | X |  |  |  |
| 1. *Were the groups comparable on demographic characteristics and clinical features?* |  | X |  | No comparison |
| 1. Was the intervention (and comparison) clearly defined? | X |  |  |  |
| 1. Was the intervention undertaken by someone experienced at performing the procedure?^1^ | X |  |  |  |
| 1. Were the staff, place, and facilities where the patients were treated appropriate for performing the procedure? (E.g. access to back-up facilities in hospital or special clinic) |  |  | X |  |
| 1. Were any of the important outcomes considered, i.e. on clinical effectiveness, cost-effectiveness, or learning curves? | X |  |  |  |
| 1. Were objective (valid and reliable) outcome measures used, including satisfaction scale? | X |  |  |  |
| 1. *Was the assessment of main outcomes blind?* |  | X |  |  |
| 1. Was follow-up long enough (>=1y) to detect important effects on outcomes of interest? |  | X |  |  |
| 1. Was information provided on non-respondents, dropouts?^2^ | X |  |  |  |
| 1. Were the withdrawals/drop-outs having similar characteristics as those completed the study and therefore unlikely to cause bias?^3^ |  |  | X |  |
| 1. *Was length of follow-up similar between comparison groups* | X |  |  |  |
| 1. Were the important prognostic factors identified, e.g. age, duration of disease, disease severity?^4^ | X |  |  |  |
| 1. *Were the analyses adjusted for confounding factors?* | X |  |  |  |

The same form was adapted to assess the quality of case series after taking out question 6, 12, 16 and 18.

Note:

1. ‘Yes’ if the practitioner received training on conducting the procedure before or conducted same kind of procedure before, i.e. no learning curve.

2. ‘No’ if participants were from those whose follow up records were available (retrospective)

3. ‘Yes’ if no withdrawal/drop out; ‘No’ if drop-out rate >=30% or differential drop-out, e.g. those having most severe disease died during follow up but the death was not due to treatment; no description of those lost.

4. ‘Yes’ if two or more than two factors were identified.

Siddiqui 2023 Date evaluated: 13/6/2023

Study ID: 10.1016/j.amjsurg.2023.04.013

| **Criteria** | **Yes** | **No** | **Unclear** | **Comments** |
| --- | --- | --- | --- | --- |
| 1. Were participants a representative sample selected from a relevant patient population, e.g. randomly selected from those seeking for treatment despite of age, duration of disease, primary or secondary disease, and severity of disease? | X |  |  |  |
| 1. Were the inclusion/exclusion criteria of participants clearly described? | X |  |  |  |
| 1. Were participants entering the study at a similar point in their disease progression, i.e. severity of disease? |  |  | X |  |
| 1. Was selection of patients consecutive? | X |  |  |  |
| 1. Was data collection undertaken prospectively? |  | X |  |  |
| 1. *Were the groups comparable on demographic characteristics and clinical features?* | X |  |  |  |
| 1. Was the intervention (and comparison) clearly defined? | X |  |  |  |
| 1. Was the intervention undertaken by someone experienced at performing the procedure?^1^ | X |  |  |  |
| 1. Were the staff, place, and facilities where the patients were treated appropriate for performing the procedure? (E.g. access to back-up facilities in hospital or special clinic) | X |  |  |  |
| 1. Were any of the important outcomes considered, i.e. on clinical effectiveness, cost-effectiveness, or learning curves? | X |  |  |  |
| 1. Were objective (valid and reliable) outcome measures used, including satisfaction scale? | X |  |  |  |
| 1. *Was the assessment of main outcomes blind?* |  | X |  |  |
| 1. Was follow-up long enough (>=1y) to detect important effects on outcomes of interest? |  |  | X |  |
| 1. Was information provided on non-respondents, dropouts?^2^ |  | X |  |  |
| 1. Were the withdrawals/drop-outs having similar characteristics as those completed the study and therefore unlikely to cause bias?^3^ |  | X |  |  |
| 1. *Was length of follow-up similar between comparison groups* |  |  | X |  |
| 1. Were the important prognostic factors identified, e.g. age, duration of disease, disease severity?^4^ | X |  |  |  |
| 1. *Were the analyses adjusted for confounding factors?* | X |  |  |  |

The same form was adapted to assess the quality of case series after taking out question 6, 12, 16 and 18.

Note:

1. ‘Yes’ if the practitioner received training on conducting the procedure before or conducted same kind of procedure before, i.e. no learning curve.

2. ‘No’ if participants were from those whose follow up records were available (retrospective)

3. ‘Yes’ if no withdrawal/drop out; ‘No’ if drop-out rate >=30% or differential drop-out, e.g. those having most severe disease died during follow up but the death was not due to treatment; no description of those lost.

4. ‘Yes’ if two or more than two factors were identified.
